# Prospects of genetics and breeding for low-phosphate tolerance: an integrated approach from soil to cell

**DOI:** 10.1007/s00122-022-04095-y

**Published:** 2022-05-07

**Authors:** Jonathan Odilón Ojeda-Rivera, Gerardo Alejo-Jacuinde, Héctor-Rogelio Nájera-González, Damar López-Arredondo

**Affiliations:** Department of Plant and Soil Science, Institute of Genomics for Crop Abiotic Stress Tolerance, Texas Tech University, Lubbock, TX USA

## Abstract

Improving phosphorus (P) crop nutrition has emerged as a key factor toward achieving a more resilient and sustainable agriculture. P is an essential nutrient for plant development and reproduction, and phosphate (Pi)-based fertilizers represent one of the pillars that sustain food production systems. To meet the global food demand, the challenge for modern agriculture is to increase food production and improve food quality in a sustainable way by significantly optimizing Pi fertilizer use efficiency. The development of genetically improved crops with higher Pi uptake and Pi-use efficiency and higher adaptability to environments with low-Pi availability will play a crucial role toward this end. In this review, we summarize the current understanding of Pi nutrition and the regulation of Pi-starvation responses in plants, and provide new perspectives on how to harness the ample repertoire of genetic mechanisms behind these adaptive responses for crop improvement. We discuss on the potential of implementing more integrative, versatile, and effective strategies by incorporating systems biology approaches and tools such as genome editing and synthetic biology. These strategies will be invaluable for producing high-yielding crops that require reduced Pi fertilizer inputs and to develop a more sustainable global agriculture.

## Introduction

Phosphorus (P) is an essential element for all living forms and one of the most restrictive nutrients for plant growth and reproduction (Ågren et al. 2012; Hou et al. 2020). Low-phosphate (Pi) availability in agricultural soils severely compromises crop productivity; thus, global P accessibility and supply have a direct implication on the food production chain worldwide. World’s population is projected to reach about 10 billion by 2050 (UN Department of Economics and Social Affairs 2015). Important challenges should be overcome to feed the world, including an increase in crop productivity from 25 to 70% without increasing agricultural land and lowering environmental pollution at the same time (Hunter et al. 2017). Favoring low-Pi tolerance in crops through improvement of Pi uptake and use efficiency will play an essential role toward developing high-yielding and better adapted plants to changing environmental conditions (Heuer et al. 2017). This represents a great challenge because of the non-renewable fate of the P reserves and the high dependency of current agricultural production systems on heavy application rates of inorganic Pi-fertilizers (Cordell and White 2015). Furthermore, the P-market economy also has a profound impact on the food production chain (Cordell et al. 2015). In this context, plant biology, biotechnology, and genetic engineering are compelled to generate innovative solutions, some of them built on traditional breeding efforts, specifically focused on improving crop varieties to reduce Pi-fertilizer inputs and enable a more sustainable agriculture.

In this review, we first discuss the role of P as an essential piece to achieve the above-mentioned goals. Then, we provide a brief description of the biological role of Pi in plants and summarize the current understanding of Pi nutrition and the regulation of Pi-starvation responses in plants. We also propose how this knowledge could be harnessed for targeted breeding of Pi uptake and use efficiency in crops. Our final purpose is to reveal the ample repertoire of genetic mechanisms behind low-Pi tolerance that can be utilized to produce low-Pi tolerant plants that have higher yield with less Pi-fertilizer input. In this review, we will define Pi-utilization efficiency as total crop dry matter production per unit of Pi applied, and uptake efficiency as the plant´s ability to obtain Pi from the soil (Wang et al. 2010).

### Phosphorus: an essential piece toward agricultural sustainability

Modern crop varieties and different inorganic Pi-fertilizers (e.g., mono- and di-ammonium-Pi, triple super-Pi, single super-Pi, and nitrogen-phosphorus-potassium (NPK)) are now widely marketed and utilized at large scale. These two components have become the pillars that sustain our society’s food production systems (Pingali 2012; John and Babu 2021). Pi-fertilizer use has increased continuously over the last decades, and its demand is projected to increase to more than 50 million tons in 2022 (FAO 2020). Forecasted demand of Pi-fertilizers is expected to increase mainly in Asia and moderately in Latin and Central America. However, no substantial increase is projected in Africa, where the Food and Agricultural Organization of the United Nations (FAO) estimated 33% of the total undernourished people in 2020 (FAO, IFAD, UNICEF, WFP, WHO 2021).

**Fig. 1:**
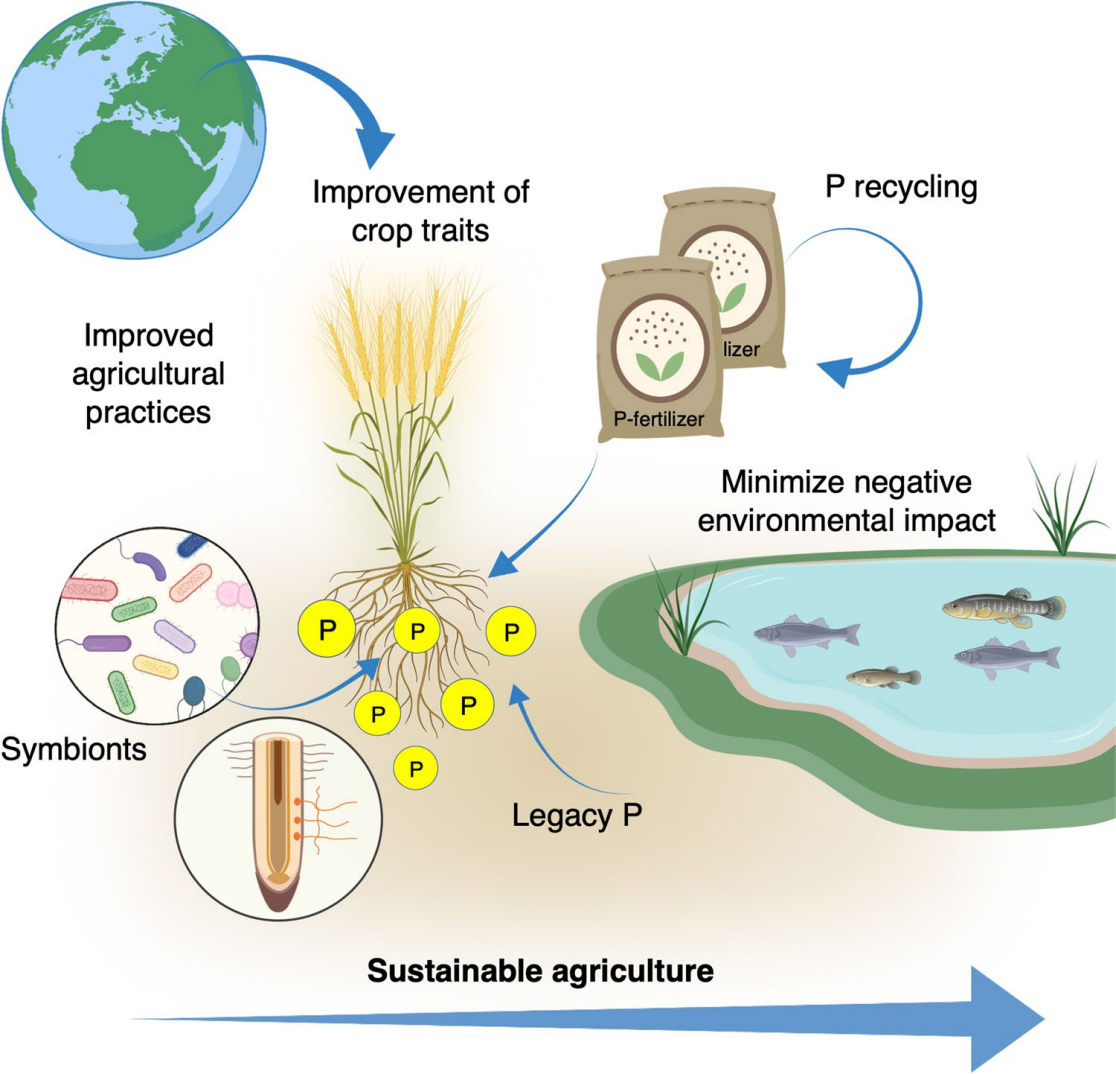
Paths toward phosphorus (P) and food security. Science-driven strategies should be implemented to be able to produce enough food to feed the growing population for the coming years. These strategies include for instance: substantial improvement of agricultural practices (i.e., fertile land and water use), development and improvement of technologies for P recycling from agricultural and industrial wastewater, deployment of symbionts, and improvement of crops traits. Improvement of plant P uptake and utilization plays a crucial role toward P and food security. Some elements in this figure were created with BioRender

Pi-rock deposits, the only source of Pi-fertilizers, are non-renewable. Therefore, a potential global P crisis has been discussed extensively during the last several years (see Cordell and White 2014). Forecasts project the potential P peak, a global Pi depletion, to occur within the next 5 to 40 decades (Mohr and Evans 2013; Van Vuuren et al. 2010; Cordell and White 2014). World resources of Pi-rock are calculated globally in over 300 billion tons of which only about 71 billion tons are considered economically extractable reserves (Jasinski 2021). Pi-rock reserves are not equally distributed, and about 90% of them are found in only six countries: Morocco and Western Sahara, China, Algeria, Syria and Brazil (Jasinski 2021). Moreover, compared to the N- and K-fertilizers market, the Pi-fertilizer market has had great fluctuation over the years suggesting that Pi supply and cost is influenced by numerous factors. It can be argued that socio-economic/political and environmental factors, rather than the geo-localization of Pi rock availability, will be the real cause of a P crisis. Nevertheless, because there are no substitutes for Pi in agriculture and the threat of Pi-limitation is imminent, humanity will unavoidably face a P crisis at some point. Science-driven, novel, and effective strategies together with inclusive and equitable environmental-social-economic policies are needed to avoid or delay the effects of a P crisis. In this context, improving the crop genetics and physiology to develop varieties that perform better under low-Pi environments becomes essential and is the focal point of this review (Fig. 1).

The challenge for modern agriculture is to produce more food and improve food quality in a sustainable way for all humans and to do so by significantly reducing dependency on Pi-based fertilizers (Fig. 1). Based on historical yield increase rates (0.9–1.6%), global production of four key crops, maize (*Zea mays*), rice (*Oryza sativa*), wheat (*Triticum aestivum*), and soybean (*Glycine max*) would be far below what is required in 2050 (Ray and Foley 2013). Thus, it is urgent to transform current food production systems. This implies implementing effective and re-oriented governance strategies which should also include ways to boost the development and introduction into the market of improved crop varieties. Improved plants, better adapted to thrive under low-Pi environments and/or more efficient in the use of Pi-fertilizers, can be generated through modern techniques such as genomic-assisted-breeding, transgenesis, and gene editing and by incorporating research and development at the systems level and synthetic biology approaches.

### Limiting factors of phosphate nutrition

P is a constituent of essential biomolecules such as nucleic acids, membrane phospholipids and ATP, and is involved in crucial biological processes. The primary source of P is the Earth’s crust, and its abundance is from 0.10 to 0.12% (on a weight basis), with most of P existing as inorganic salts like calcium (Ca)-, potassium (K)- and aluminum (Al)-Pi, and to a lesser extend as Pi-organic compounds (Mackey and Paytan 2009). Plants take up Pi from the soil solution via membrane transporters and then reach a concentration of up to 100 mg L^−1^ in the xylem sap and around 4000 mg kg^−1^ in the seed (Tiessen 2008; Versaw and Garcia 2017), indicating that adequate Pi nutrition is required for maintaining crop productivity. At a first level, physiological and metabolic mechanisms allow the plant to cope with both excessive and deficient Pi levels so it can thrive under either condition. When Pi levels are too high, the plant prevents Pi toxicity by increasing Pi efflux and storage in the vacuoles and reducing the activity of high affinity Pi transporters (Schachtman et al. 1998). When Pi is low, the plant activates a series of mechanisms including the activation of a high affinity Pi transporter system, the remobilization and recycling of Pi from non-essential molecules and an enhanced interaction with the rhizosphere to take advantage of dissolved Pi. All these mechanisms constitute the general response of the plant to low-Pi availability and will be explained in more detail in the following sections.

**Fig. 2:**
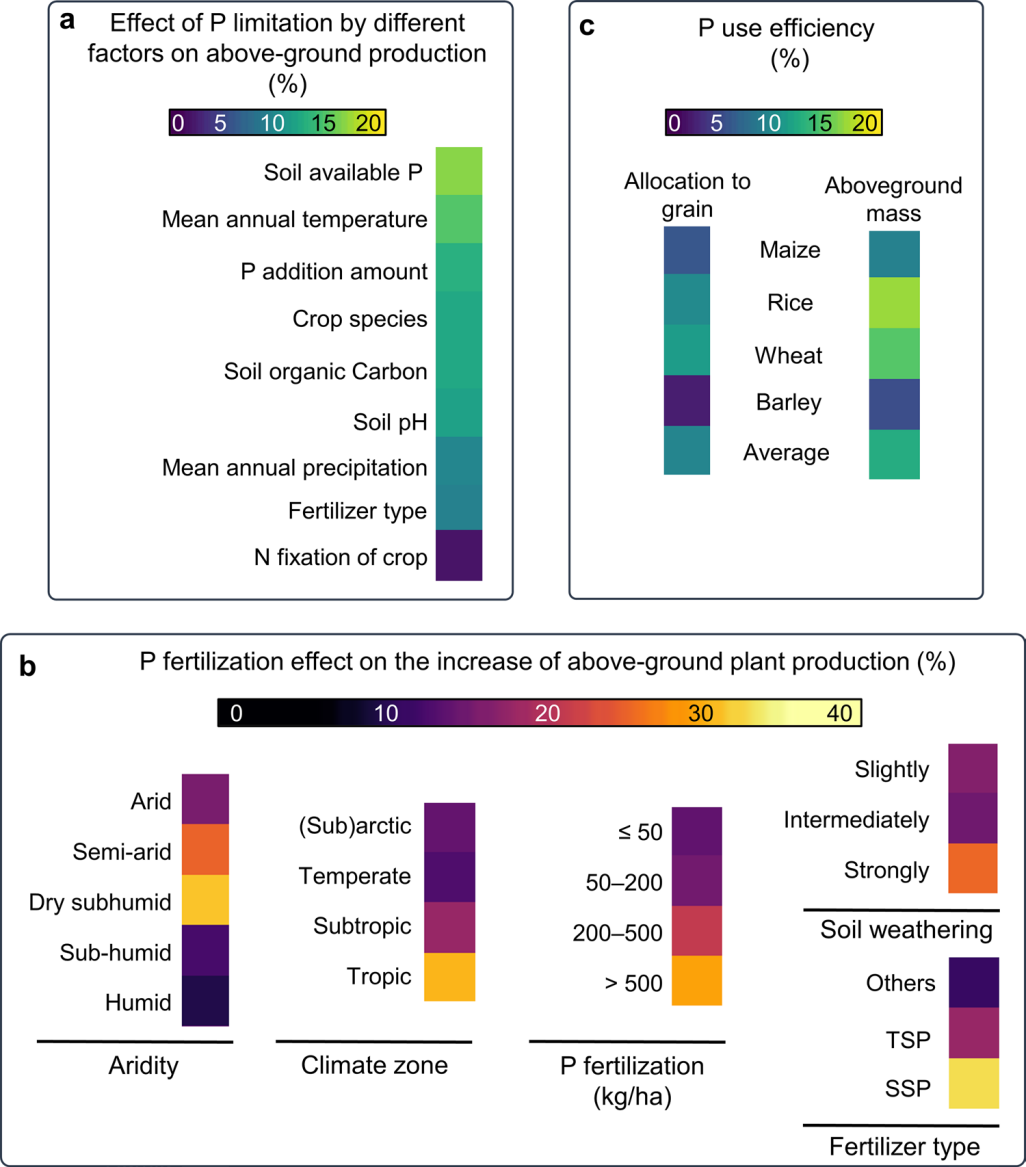
Current phosphorus (P) fertilization practices are largely inefficient and determined by several factors besides the P fertilizer amount applied to soil. Heatmap illustrating **a** the size of the effect that the listed factors can have in limiting the variance of above-ground crop production; **b** the effect that P fertilization can have in increasing aboveground plant production in function of the properties of the cropland (aridity, climate zone, soil weathering) and the P fertilization regime (amount and fertilizer type); and **c** P use efficiency in common cereals. P fertilizer use efficiency for grain (left) is calculated as follows: (P content in grain with P fertilization—P content in grain without P fertilization)/P fertilizer amount applied × 100%. P fertilizer use efficiency for above-ground mass production (right) was calculated as follows (P content in aboveground biomass with P fertilizer—P content in aboveground biomass without P fertilizer)/P fertilizer amount × 100%. **a, b** Heatmaps are based on the data published by Hou et al. (2020). The heatmap presented in (**c**) is based on the data published by Yu et al. (2021). Heat maps were prepared using ComplexHeatmap R package (Gu et al. 2016b)

There are numerous external factors influencing Pi availability and plant uptake rates, including: Pi chemical properties, soil properties (pH, cations), environmental conditions, and agricultural practices. For example, soil Pi availability is limited because Pi is very susceptible to adsorption and precipitation making Pi unavailable for plant uptake (Yadav et al. 2012). Furthermore, cations such as Ca^2^^+^, Al^3^^+^, and Mg^2^^+^ strongly interact with Pi, limiting even more its availability. Another factor contributing to low-Pi availability is soil erosion (Alewell et al. 2020). A heatmap that summarizes the main factors limiting Pi availability and their estimated influence on aboveground plant productivity is presented in Fig. 2a (based on the data analysis provided in Hou et al. 2020 and Yu et al. 2021). Because all these factors influence Pi-fertilization efficiency, a heatmap summarizing their effect on Pi-fertilization efficiency and its consequences on aboveground plant production is presented in Fig. 2b. It is worth mentioning that the most important cereal crops including maize, rice, wheat, and barley (*Hordeum vulgare*) have low-Pi fertilizer use efficiency which is in average less than 10% (Fig. 2c; Yu et al. 2021).

After the plant has taken up Pi from the soil, the remaining Pi gets fixed into soil particles and becomes part of the so-called pool of residual P (difference between P inputs and P outputs) or legacy P (Pavinato et al. 2020). Importantly, large fractions of legacy P can be made naturally available, i.e., by changes in soil properties either caused by the climate conditions, the plant and/or its associated microbiome, and further removed and taken up in subsequent years (Khan et al. 2007; Sharpley et al. 2013; Pavinato et al. 2020). Therefore, legacy P might play an essential role in increasing or at least maintaining crop productivity and decreasing Pi-fertilizers use in the near future (Sattari et al. 2012; Alewell et al. 2020). Interestingly, a recent study suggests that increasing Pi-fertilization by 7% in the Pi-depleted soils in sub-Sahara Africa would boosts crop production and contribute to meet the global food demand by 2050, and thereby, decrease cropland expansion (Mogollón et al. 2021). The latter strategy should be undoubtedly accompanied by improved crops provided with adequate traits to succeed under such adverse environmental growth conditions.

## Plant adaptive mechanisms associated with low-phosphate conditions

Several crops and model plants have served to study plant responses to Pi-deprivation at different levels (i.e., metabolic, physiological and molecular). This has broadened our understanding of metabolic and organ plasticity in response to Pi-starvation, Pi-sensing mechanisms, and how key molecular regulators trigger adaptive molecular responses. Plant molecular responses to Pi-availability can be categorized into two types of responses: local and systemic responses (Péret et al. 2011). Local responses depend on the external concentrations of Pi that the root is in contact with and modulate modifications of root architecture and the exudation of anions of organic acids mainly malate and citrate (Raghothama and Karthikeyan 2005; Plaxton and Tran 2011; Péret et al. 2011). Systemic responses comprehend the activation of physiologic, metabolic, and molecular adjustments at the whole plant level when internal Pi homeostasis is compromised. However, evidence from transcriptomic analysis of mutants defective in the local response to low-Pi indicates that local and systemic responses are not completely independent and there is some degree of crosstalk between these two signaling pathways (Mora-Macías et al. 2017; Raya-González et al. 2021). However, this dissection provides a framework that we will use to explain and summarize findings on both the plant adaptive mechanisms, and the molecular components that sense and control responses to internal and external Pi levels, and how these regulatory modules can be used to improve low-Pi tolerance.

### Systemic responses

#### Phosphate uptake and translocation

Pi is generally required at a higher concentration inside the plant cell (5–10 mM) than it is in the soil solution in which it seldom exceeds 10 μM (Raghothama and Karthikeyan 2005; Marschner 2011). Thus, it can be actively taken up against a concentration gradient by Pi transporter proteins present in the plasma membrane of epidermal root cells. Pi is then distributed symplastically across the root to reach the xylem and the aerial parts of the plant (López-Arredondo et al. 2014; Malhotra et al. 2018). Two types of Pi transporter proteins enable Pi acquisition: low-affinity Pi transporters with affinity constant (K_m_) values in the 50–300 μM range, and high-affinity Pi transporters with a K_m_ in the 3–10 μM range (Raghothama 1999; Hasan et al. 2016). High-affinity Pi transporters in plants are encoded by *PHOSPHATE TRANSPORTER* (*PHT)* genes which are phylogenetically classified into five families, *PHT1-5* (Wang et al. 2017), from which the *PHT1* is known to be mainly involved in root-mediated Pi uptake from soil. *PHT1* genes are systemically induced in *Arabidopsis* under Pi-limiting conditions (Raghothama and Karthikeyan 2005; Thibaud et al. 2010). PHT1 transporters have been characterized in *Arabidopsis* and several crops such as maize, rice, wheat, soybean, tomato (*Solanum lycopersicum*), and barley (for review see Wang et al. 2017). In the case of *Arabidopsis*, AtPHT1;1 and AtPHT1;4 account for up to 75% of total plant Pi uptake (Shin et al. 2004). In rice, OsPHT1;4 is the main transporter involved in root-mediated Pi uptake and its overexpression leads to Pi over-accumulation in roots (Ye et al. 2015). Moreover, maize *ZmPHT1* genes are induced by Pi starvation and mediate the symbiotic association with arbuscular mycorrhizal fungi (AMF). Phosphate transporters upregulated by AMF in rice (OsPT11/13) and soybean (GmPT7/10/11) support a role for plant Pi-uptake at this symbiotic interface (Tamura et al. 2012; Yang et al. 2012).

The root cap, which stands at the forefront of soil exploration, has been described to enable up to 20% of the total plant Pi uptake. Interestingly, *AtPHT1;4* is predominantly expressed in this region of the root, and root-cap-mediated Pi-uptake was also reported to occur in rice and lotus (Kanno et al. 2016), indicating that this Pi-uptake mechanism might be generally present in plants. Proteins that belong to the PHT1-5 families of Pi transporters play additional roles involved in maintaining Pi homeostasis, either by facilitating Pi-uptake or by enabling remobilization of internal Pi among different tissues or organelles. This is particularly important because most of the Pi in the cell is stored in the vacuole, while only 1–5% is present in the cytoplasm (Wang et al. 2017; Versaw and Garcia 2017). Once Pi is taken up by the root cells, root-to-shoot translocation of Pi is enabled by PHOSPHATE1 (PHO1), AtPHO1 in *Arabidopsis*, a protein involved in loading Pi into the xylem (Poirier et al. 1991; Hamburger et al. 2002). Orthologs for PHO1 have been identified and characterized to have similar functions in soybean, rice, and maize (Secco et al. 2010; Salazar-Vidal et al. 2016; Wang et al. 2019c).

#### Phosphate scavenging and remobilization

Besides the induction of high affinity Pi transport and translocation in response to low-Pi, systemic responses in plants also comprehend the activation of Pi remobilization, scavenging and recycling mechanisms. Plants recycle Pi from the hydrolysis of phospholipids which leads to an increase in internal Pi availability (Plaxton and Tran 2011); non-Pi lipids like galactolipids and sulfolipids replace phospholipids to maintain the functionality and structure of the plasma membrane. Enzymes in *Arabidopsis* that are involved in this process include PHOSPHOLIPASE D ZETA 2 (AtPLDZ2) and MONOGALACTOSYL DIACYLGLICEROL SYNTHASE 1–2 (AtMGDG1-2) and DIACYLGLICEROL SYNTHASE 1–2 (AtDGDG1-2) and SULFOQUINOVOSYL DIACYLGLYCEROL SYNTHASE 1 (AtSQD1) (Essigmann et al. 1998; Awai et al. 2001; Kelly et al. 2003; Cruz-Ramírez et al. 2006; Plaxton and Tran 2011). As indicated by the names of these enzymes, they play roles in phospholipid breakdown and the synthesis of substitute monogalactosyl-, digalactosyl-, sulfoquinovosyl diacylglycerol-, and glucuronosyl diacylglycerol-lipids (Okazaki et al. 2013). Phospholipid hydrolysis and Pi-recycling from nucleic acids, through the induction of nuclease-coding genes, contribute to buffering the cytosolic Pi pools when systemic levels of Pi are limiting (Plaxton and Tran 2011; Jeong et al. 2017). Maintaining adequate cytosolic Pi levels is crucial because low Pi levels have a negative effect on photosynthesis which eventually leads to the inhibition of plant growth and development (Ajmera et al. 2019). Interestingly, plants belonging to the Proteaceae family predominantly use galactolipids and sulfolipids instead of phospholipids in mature photosynthetic leaves allowing them to maintain high photosynthetic Pi-use efficiency and thrive on the sands of southwestern Australia (Hayes et al. 2018).

Pi scavenging in plants is promoted through the upregulation of genes encoding acid phosphatases (AP) which hydrolyze Pi from organic Pi-esters present in a wide variety of organic compounds including nucleic acids, ATP, 3-phosphoglycerate, and various hexose-Pi compounds (Hurley et al. 2010). A large group of these proteins, named purple acid phosphatases (PAPs), is induced as part of the systemic response to Pi-starvation and help the plant in Pi-solubilization from organic Pi sources (Hurley et al. 2010; Thibaud et al. 2010). In *Arabidopsis*, AtPAP26 is the predominantly secreted and intracellular phosphatase (Li et al. 2002; Hurley et al. 2010). AtPAP10 and AtPAP12 are not secreted, but associated with the root surface, increasing the availability of Pi where it is useful for the plant (Tran et al. 2010; Wang et al. 2011). Vacuolar or other intercellular PAPs are expressed in stage-specific and tissue-specific fashion to mobilize Pi from storage organelles or from senescent leaves (Gao et al. 2017). Putative PAPs have been identified in maize (González-Muñoz et al. 2015), soybean (Li et al. 2012), and rice (*Oryza sativa*, (Zhang et al. 2011). Enhanced levels of PAPs remains an interesting perspective to boost Pi uptake in crops.

### Local monitoring of external phosphate

Local responses to low-Pi levels include modifications in root development and the subsequent remodeling of root system morphology and architecture in response to the concentrations of external Pi. These responses become important because plant Pi-uptake capacity is greatly increased by an enhancement in the root’s ability to explore the soil which is accompanied by higher expression of Pi-transporter and phosphatase genes and an increase in exudation of organic acids from root tips (Sánchez-Calderón et al. 2005; Ruiz Herrera et al. 2015). Earliest evidence of these type of responses was reported in common bean and subsequently in *Arabidopsis* (Lynch and Brown 2001; López-Bucio et al. 2002).

Root architecture modifications are oriented to the development of a shallow root architecture in order to more efficiently explore the top layers of the soil where Pi tends to accumulate. This adaptation is known as topsoil Pi foraging (Lynch and Brown 2001) and is common to multiple plant species, including cereals that do not form a main root growth axis (Lynch and Brown 2001; Zhu et al. 2005; see Péret et al. 2014 for review). Depending on the species, modifications of root architecture and morphology also include an increase in the density and length of root hairs (Bates and Lynch 1996), an increase in the emergence of lateral roots with a shallower root angle (Lynch and Brown 2001; López-Bucio et al. 2002), the formation of adventitious roots (Ochoa et al. 2006), and the inhibition of primary root growth (Gutiérrez-Alanís et al. 2018). Additionally, there are less common but important root morphological adaptations that are triggered in response to limited Pi-availability, such as the formation of dense interspaced clusters of roots called ‘cluster roots’. These structures are associated with plants adapted to environments with extreme Pi-scarcity like the Proteaceae and the Cyperaceae (Lambers et al. 2012; Ruiz Herrera et al. 2015). Characteristically, carboxylates, mainly citrate, are profusely secreted through these structures to solubilize Pi from both inorganic and organic Pi-compounds present in the soil (Lambers et al. 2012; Ruiz Herrera et al. 2015).

These responses are regulated by local signals, at least at the transcriptional level, independently of the systemic Pi homeostasis status of the plant (Thibaud et al. 2010; Péret et al. 2011) and are determined by the dynamics of different hormones. Low external Pi increases the root sensitivity to auxins which has been described as a key component regulating lateral root growth (López-Bucio et al. 2002). An increase in lateral root formation due to increased mitotic activity is observed during Pi-deprivation in *Arabidopsis* (Ajmera et al. 2019), which is regulated by the auxin receptor TIR1 and the transcription factors ARF7/19 (Auxin Response Factor 7 and 19) (Pérez-Torres et al. 2008).

## Genetic regulation of low-phosphate adaptations

### Regulons controlling phosphate homeostasis

Systemic transcriptional responses to Pi-deprivation are largely controlled by the MYB transcription factor PHOSPHATE STARVATION RESPONSE 1 (PHR1) which was first discovered in *Arabidopsis* (Rubio et al. 2001). Pi deprived *phr1* seedlings do not accumulate anthocyanins, which is characteristic of Pi-deprived plants, and present low expression of phosphate starvation responsive (PSR) genes (Rubio et al. 2001; Bustos et al. 2010). Interestingly, *phr1* mutants do not present defects in the root developmental response to low-Pi, which provides evidence of the dissection between local and systemic responses to Pi-deprivation (Rubio et al. 2001). AtPHR1 regulates the expression of PSR genes by binding to the imperfect palindromic sequence GNATATNC that is known as PHR1-Binding Site (P1BS), which is present in the promoter sequences of its target genes (Bustos et al. 2010). AtPHR1 has orthologs that play a similar function in several crop species including maize ZmPHR1 (Calderón-Vázquez et al. 2011), rice OsPHR2 (Zhou et al. 2008), common bean PvPHR1 (Valdés-López et al. 2008) and wheat TaPHR1 (Wang et al. 2013).

**Fig. 3:**
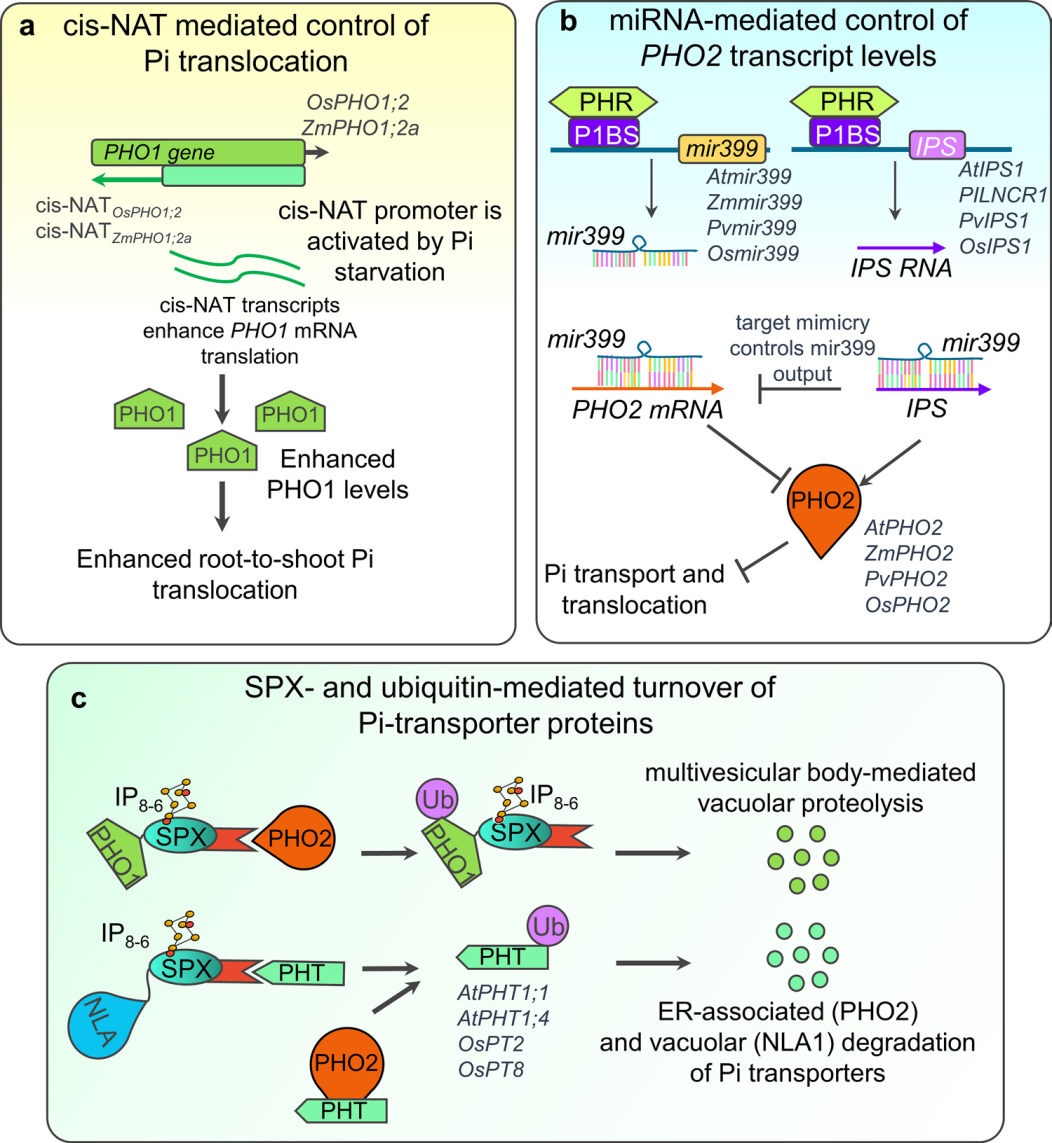
Regulons controlling plant phosphate (Pi) homeostasis. Schematic summary of **a** cis-NAT mediated control of Pi translocation; **b** miRNA-mediated control of *PHO2* transcript levels; and **c** ubiquitin-mediated turnover of Pi-transporter proteins in plants. Explanation and discovery of the regulons for *Arabidopsis* and crops is presented throughout the text

When Pi levels are limiting, PHR1 transcription factors upregulate the expression of high affinity PHT1-like genes (Nussaume et al. 2011). Pi transport is followed by root-to-shoot translocation enabled by PHO1. However, *OsPHO1;2* expression is not responsive to Pi-deprivation which suggests post-transcriptional activation mechanisms (Secco et al. 2010). Interestingly, the cis-Natural Antisense Transcript (cis-NAT) *cis-NAT*_*PHO1;2*_ present in the *OsPHO1;2* locus is induced in response to Pi-deficiency and stimulates PHO1;2 accumulation by enhancing its translation independently of *PHO1;2* mRNA levels (Jabnoune et al. 2013). The cis-NAT_*PHO1;2a*_ was also found out to be present in one of the four *PHO1* orthologs in maize, *ZmPHO1;2a*, and responsive to Pi-starvation (Salazar-Vidal et al. 2016), suggesting that its upregulation might play a similar role. cis-NAT regulation of ZmPHO1 translation (Fig. 3a) emerges as an interesting perspective for studies, engineering, and breeding of Pi homeostasis and Pi translocation in crops.

PHOSPHATE2 (PHO2), a ubiquitin E2-ligase, plays also a key role in Pi homeostasis because it regulates the ubiquitin-mediated degradation of Pi-transporter proteins PHT1-4 and the phosphate translocator PHO1, thus preventing Pi shoot toxicity (Liu et al. 2012; Huang et al. 2013). Under Pi-starvation conditions, PHR1 upregulates the expression of the microRNA *Atmir399* which triggers the downregulation of AtPHO2 in *Arabidopsis* (Bari et al. 2006; Hsieh et al. 2009; Castrillo et al. 2017). Atmir399-output is also modulated by AtIPS1, a long noncoding RNA (lncRNA) also induced by AtPHR1, which sequesters Atmir399 to prevent cleavage of PHO2 mRNA (Franco-Zorrilla et al. 2007). The discovery of this regulon revealed target mimicry as a biological mechanism to inhibit miRNA activity (Franco-Zorrilla et al. 2007). There is conservation of the PHR1-mir399-IPS1 regulon in various crops including maize (ZmPHR1-Zmmir399- PILNCR19), rice (OsPHR2-Osmir399-OsIPS1/2), and common bean (PvPHR1-Pvmir399-PvIPS1*)* (Valdés-López et al. 2008; Hu et al. 2011; Du et al. 2018). A schematic summary of miRNA-mediated control of *PHO2* transcript levels in plants is presented in Fig. 3b.

Another ubiquitin-ligase that controls Pi homeostasis in plants is *NITROGEN LIMITATION ADAPTATION 1* (NLA) which activates the degradation of PHT1 Pi-transporters via ubiquitin-mediated endocytosis in *Arabidopsis* (Lin et al. 2013) and rice (Yue et al. 2017). AtNLA1 is negatively regulated by both Atmir399 and Atmir827 microRNAs which are induced upon Pi-starvation (Kant et al. 2011). Even though both NLA1 and PHO2 regulate turnover of Pi-transporters, they have been reported to act independently as they do not interact with each other (Lin et al. 2013; Yue et al. 2017). Both NLA1 and PHO2 prevent Pi-toxicity when Pi levels are high. A schematic summary of ubiquitin-mediated turnover of Pi-transporter proteins in plants is presented in Fig. 3c.

### Regulation mechanisms by inositol polyphosphate sensing

**Fig. 4:**
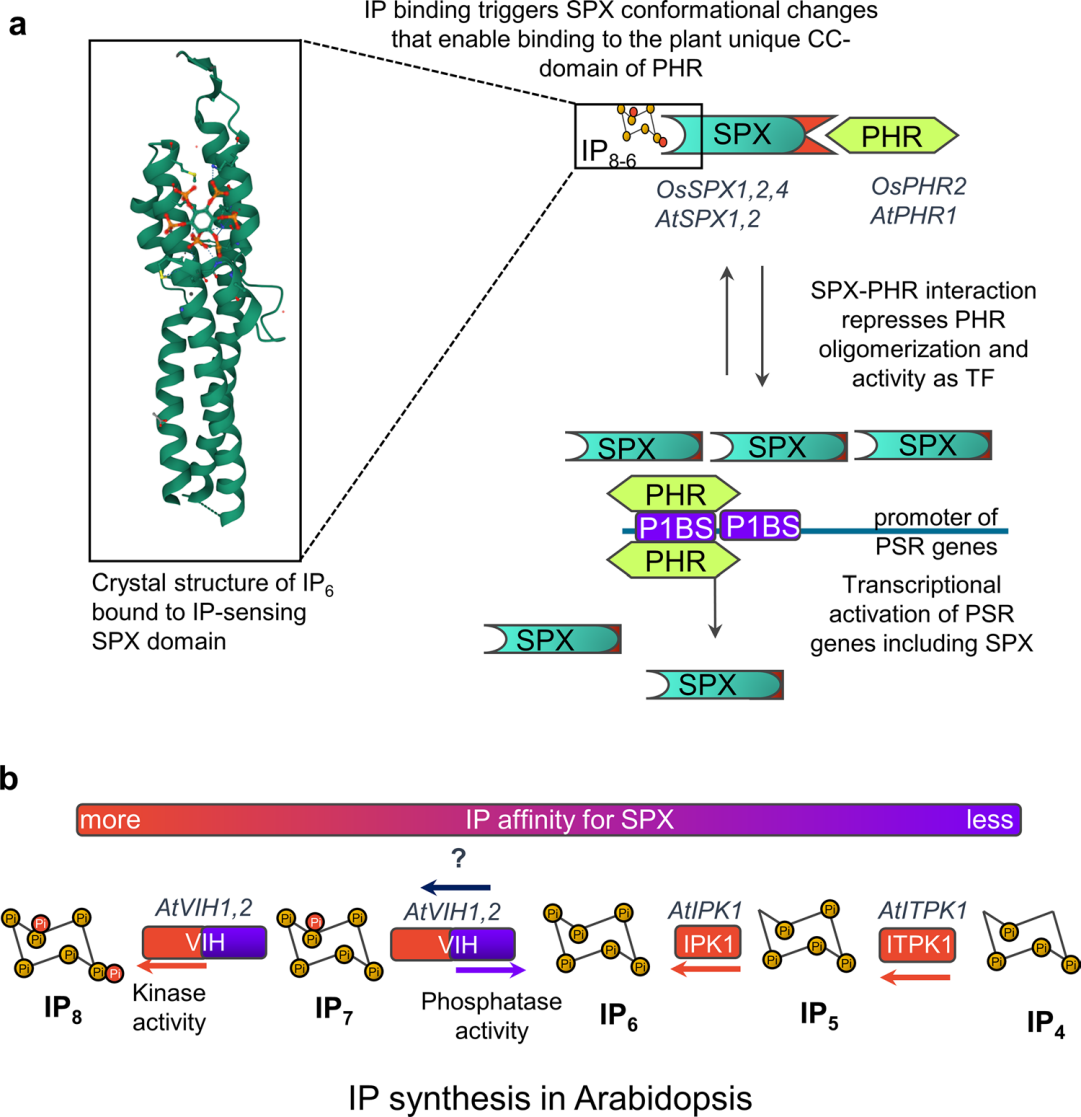
Control of plant phosphate (Pi) homeostasis by inositol (IP)-signaling and IP-sensing domains. **a** Schematic representation of SPX-domain regulation on plant PHR transcription factors. See main text for complete explanation. Because SPX-domains are IP-sensing domains conserved among eukaryotes (Wild et al. 2016), the crystal structure of IP_6_-bound SPX domain from the fungi *Chaetomium thermophilum* is presented as an example. **b** Representation of IP synthesis in plants. Dual phosphatase/pyrophosphate kinases AtVIH1 and AtVIH2 phosphorylate InsP_7_ to produce InsP_8_ and dephosphorylate InsP_7_ to InsP_6_. It is currently unknown which enzyme catalyzes the formation of InsP_7_. AtIPK1 and AtITPK1 kinases catalyze, respectively, the subsequent phosphorylation of IP_4_ to IP_5_ and to further IP_6_

Because PHR1 transcription factors play a central role in activating the expression of multiple genes that enable plant adaptation to Pi-scarcity, an interesting question regarding Pi sensing is how PHR1 transcription factors are regulated. This process is known to be under control of SPX (SIG1-Pho81-XPR1) regulatory proteins in *Arabidopsis* and rice (Puga et al. 2014; Wang et al. 2014). Earliest evidence demonstrated that SPX1 binds to AtPHR1 in plants grown under optimal Pi conditions inhibiting its activity as a transcription factor (Puga et al. 2014). Interestingly, AtPHR1 induces *AtSPX1* expression when Pi levels are limiting, forming a negative regulatory loop that was proposed to enable the plant to rapidly turn off PHR1 activity upon Pi refeeding or a sudden increase in cellular Pi levels (Fig. 4a). The PHR1-SPX1 regulon was simultaneously reported in rice in which OsSPX1 and OsSPX2 were shown to interact with OsPHR2 and inhibit its binding to P1BS sites in a Pi-supply dependent fashion (Wang et al. 2014). AtPHR1-SPX1 and OsPHR2-SPX1-2 regulons provided the first example of a Pi-sensing mechanism in plants. Further investigation of SPX-domain proteins in rice and *Arabidopsis* demonstrated that they are cellular sensors of inositol-polyphosphate (IP) levels (Fig. 4a). SPX-domain proteins selectively interact with target transcription factors in the presence of IP molecules in the µm concentration range (~ 20 µM) and with high affinity, i.e., K_d_ of 50 and 7 µM for IP_6_ and IP_7_, respectively (Wild et al. 2016).

Besides their role in regulating transcription factor activity, SPX-regulatory proteins play additional roles in the regulation of Pi homeostasis. Examples of these proteins include the previously mentioned Pi translocator PHO1 and NLA1. AtNLA1 and OsNLA1 interact with Pi transporters via their SPX domain (Lin et al. 2013; Yue et al. 2017). In the case of AtPHO1, the function of the SPX-domain is still unknown, but it is involved in regulating AtPHO1 Pi-translocation activity (Wege et al. 2016) (Fig. 3c). Because SPX-domain containing proteins have been identified and functionally validated in some crops in addition to rice (see Liu et al. 2018b for review), their study and manipulation open an interesting perspective for breeding approaches to generate low-Pi tolerant crop varieties.

Recent studies have focused on the nature of the IP molecule that regulates Pi homeostasis through the SPX-PHR regulon. Two independent reports proposed that the enzymes AtVIH1 and AtVIH2 play a key role in controlling plant Pi homeostasis by regulating the turnover of IP_8_ inside the plant cell (Zhu et al. 2019; Dong et al. 2019). *Atvih1vih2* double mutants have undetectable levels of IP_8_ and constitutive activation of PSR genes under high-Pi conditions (Zhu et al. 2019; Dong et al. 2019). IP_8_ levels were shown to correlate with the intracellular concentration of Pi, supporting the role of IP_8_ as a Pi-homeostasis signaling molecule in *Arabidopsis* (Zhu et al. 2019; Dong et al. 2019). Interestingly, *Arabidopsis Atipk1* and *Atitpk1* mutants which are defective in the synthesis of IP_5_ and IP_6_ are also altered in Pi homeostasis (Kuo et al. 2018). These mutants present higher shoot Pi content, higher expression of Pi-transporter genes and other transcripts regulated by AtPHR1, and higher Pi-uptake relative to wild-type plants under Pi-sufficiency conditions (Kuo et al. 2018). These data suggest that specific species of IP might play a specific role in the regulation of plant Pi homeostasis and that it is not solely controlled by IP_8_. In support of this notion, a recent report of novel alleles of *Atvih1* and *Atvih2* double mutants showed that IP_8_ levels are downregulated in these mutants; however, the expression of AtPHR1-targets remains responsive to Pi-deprivation (Land et al. 2021). Because IP_6_, IP_7_, and IP_8_ can bind the SPX domain (Wild et al. 2016), further *in planta* studies on the specific roles of IPs individual species are required. A schematic summary of IP synthesis in *Arabidopsis* is presented in Fig. 4b.

### Epigenetic regulation mechanisms activated under phosphate starvation

Epigenetic regulation is important because changes in chromatin structure and DNA modifications influence plant adaptation to stress conditions (Grimanelli and Roudier 2013; Zhang et al. 2018a; Séré and Martin 2019). Whole genome cytosine DNA methylation (mC) dynamics respond to Pi-availability in multiple plant species including tomato, soybean, rice and *Arabidopsis* (Secco et al. 2015; Yong-Villalobos et al. 2015; Chu et al. 2020; Tian et al. 2021). *Arabidopsis* mutants defective in mC present disrupted responses to low-Pi availability including increased lateral root density, lower phosphatase activity and reduced total P content (Yong-Villalobos et al. 2015). Moreover, global mC changes in *Arabidopsis* and soybean correlate with positive and negative changes in gene expression depending on the mC pattern (Yong-Villalobos et al. 2015; Chu et al. 2020). However, in the case of tomato and rice, Pi-deprivation induced changes in global mC patterns do not correlate with global changes in gene expression (Secco et al. 2015; Tian et al. 2021). Therefore, a clear role for global mC dynamics on the genome-wide gene expression regulation during plant Pi-starvation has not been clearly established, rather it seems that methylation occurs only for a specific set of Pi-starvation responsive genes and that this might take place in tissue-specific fashion (Secco et al. 2015; Yong-Villalobos et al. 2015). Interestingly, data indicate that transposable elements are enriched in mC in response to Pi-limitation, which has been suggested to protect genome stability (Secco et al. 2015; Yong-Villalobos et al. 2015; Chu et al. 2020; Tian et al. 2021). Thus, methylation-directed transposable element silencing seems to be a conserved mechanism among multiple plant species. Nonetheless, more studies are required to further understand additional roles of mC in plant adaptation to low-Pi conditions.

Another epigenetic regulatory mechanism of gene expression is the modification of chromatin accessibility (Kornberg and Lorch 2003; Mellor 2005). Pi-deprived root cells undergo genome-wide changes of chromatin accessibility in *Arabidopsis* and AtPHR1 plays a key role in this process (Barragán-Rosillo et al. 2021), providing yet another role for PHR1. Furthermore, up to 40% of rice genes undergo chromatin state changes in response to low-Pi stress which positively correlates with differentially expressed genes (Foroozani et al. 2020). Another chromatin modification, histone deacetylation, represses the transcriptional activation of genes related to the modification of root architecture in response to low-Pi in *Arabidopsis* (Xu et al. 2020). Altogether, recent evidence highlights the importance of epigenetic regulation on plant adaptation to low-Pi availability and its value as the foundation for the development of innovative genome manipulation tools that might enable breeders to produce low-Pi tolerant crops.

### Control of root changes

**Fig. 5:**
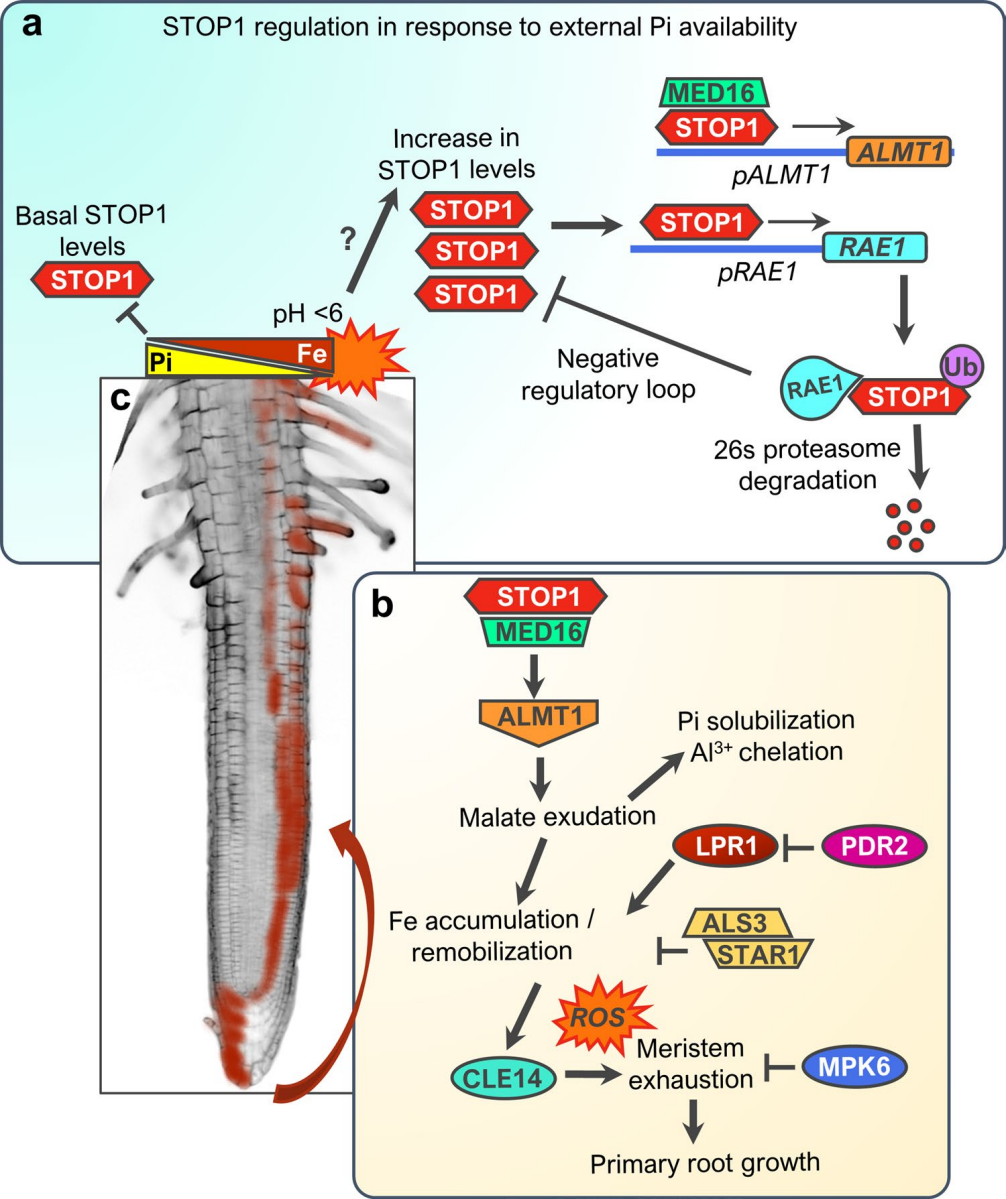
Local phosphate (Pi) sensing in the *Arabidopsis* root tip. **a** STOP1 accumulates in response to root tip contact with low Pi/Fe ratio and pH < 6; it regulates its own turnover via activation of *RAE1/RAH1*. MEDIATOR subunit MED16 promotes the STOP1-activation of *ALMT1* expression under low-Pi conditions. **b** STOP1 activates *ALMT1* expression which results in the increase of malate exudation to the root apoplast contributing to Pi solubilization, Al chelation, and Fe accumulation/remobilization in the root apical meristem. STOP1-ALMT1 and PDR2-LPR1 modules are essential and cooperate independently for this phenomenon. The tonoplast transporter complex ALS3/STAR1 is involved in the negative regulation of Fe accumulation. MPK6 kinase negatively regulates meristem maintenance in response to the low Pi/Fe ratio. Fe remobilization activates *CLE14* expression which leads to the downregulation of meristem maintenance pathways. **c** Schematic of Fe remobilization in the root meristem (see text for explanation)

The regulation of primary root growth in response to external Pi availability in *Arabidopsis* is one of the best characterized developmental programs (for reviews see Abel 2017; Gutiérrez-Alanís et al. 2018). Upon contact of the root tip with low-Pi medium, a determinate developmental program known as meristematic exhaustion is activated in the root apical meristem (Sánchez-Calderón et al. 2005; Svistoonoff et al. 2007). Meristematic exhaustion consists in a cease in cellular proliferation and the premature differentiation of the cells that comprise the root meristem (Sánchez-Calderón et al. 2005). Studies have highlighted a central role for the transcription factor SENSITIVE TO PROTON RHIZOTOXICITY 1 (AtSTOP1) and iron (Fe) signaling in this developmental program (Fig. 5a, b). Pi and Fe antagonistically interact to limit each other’s availability, and this external Pi/Fe ratio influences meristematic activity in the root (Müller et al. 2015). The levels of STOP1 increase in response to low Pi/Fe ratio which induces its target gene *ALUMINUM ACTIVATED MALATE TRANSPORTER 1* (*AtALMT1)* (Balzergue et al. 2017; Mora-Macías et al. 2017; Godon et al. 2019)*.* The AtALMT1 protein facilitates malate efflux into the root apoplast which together with the activities of the multicopper oxidase LOW PHOSPHATE ROOT 1 (AtLPR1) and the PHOSPHATE DEFICIENT ROOT 2 (AtPDR2) P5-type ATPase, adjust root meristem activity in response to external Pi (Ticconi et al. 2009; Müller et al. 2015). AtALMT1, AtPDR2 and AtLPR1 activities contribute to changes in zone-specific Fe accumulation in response to low-Pi availability, referred to as Fe remobilization (Fig. 5c). Fe remobilization activates the expression of *CLAVATA3/EMBRYO SURROUNDING REGION 14 (AtCLE14)* signaling peptide which is perceived by AtCLAVATA2/AtPEPR2 receptors, then triggering the downregulation of auxin and other signaling pathways related to meristem maintenance and ultimately leading to the inhibition of primary root growth (Gutiérrez-Alanís et al. 2017). A mitogen-activated protein kinase (MPK), AtMPK6, has been recently linked to the determination of primary root growth by integrating Pi/Fe signals to adjust cellular divisions through auxin signaling in the meristem (López-Bucio et al. 2019). However, the molecular components that are targets of MPK6-dependent regulation remain unknown, and it is yet to be determined whether MPK6 interacts with some of the components that adjust root growth in response to low-Pi availability.

The molecular nature of the mechanism that activates the initial spike in STOP1 accumulation leading to determinate root growth remains unknown. A recent report showed that the activation of *ALMT1* expression by STOP1 is facilitated by a subunit of the MEDIATOR complex, AtMEDIATOR16, which also acts as a transcriptional co-activator of several low-Pi responsive genes, including *AtALMT1* (Raya-González et al. 2021)*.* Following its accumulation, STOP1 turnover is controlled by ubiquitin ligases REGULATION OF ALMT1 EXPRESSION 1 (RAE1) and its homolog RAE1 HOMOLOG 1 (RAH1) that tag STOP1 for 26S  proteasomal degradation (Zhang et al. 2019; Fang et al. 2021). Because *RAE1/RAH1* are targets of STOP1, this constitutes a regulation loop by which STOP1 controls its own turnover (Fig. 5b). Other components involved in the regulation of primary root growth include *ALUMINUM SENSITIVE 3* (*ALS3)* and *SENSITIVE TO ALUMINUM RHIZOTOXICITY* (*STAR1*) (Dong et al. 2017). A genetic dissection of responses to low-Pi proposed that the ALS3/STAR1 complex functions upstream of STOP1 because STOP1 accumulates more in *als3* mutant than in wild-type seedlings under high and low-Pi conditions, whreas *als3stop1* mutants continue primary root growth under Pi-starvation (Wang et al. 2019a, b, c). However, because there is evidence that *ALS3* is a STOP1 target (Sawaki et al. 2009; O’Malley et al. 2016; Ojeda-Rivera et al. 2020), at least two negative regulation feedback loops (RAE1/STOP1, ALS3/STOP1) control STOP1 turnover in response to abiotic stress factors.

*AtSTOP1* and *AtALMT1* were first described to contribute to *Arabidopsis* tolerance to acidic pH and increased Al levels that prevails in acidic soils by activating and mediating, respectively, organic acid exudation (Hoekenga et al. 2006; Iuchi et al. 2007). STOP1-mediated organic acid exudation plays multiple beneficial roles including the prevention of detrimental effects of Al in root development, the release of Pi from complexes with cations for root uptake and the modification of root growth. Al-resistance related loci might be of the outmost interest for breeding crop varieties with enhanced root morphology for topsoil exploration in acidic soils. A recent transcriptional dissection of root responses under this condition pinpoints some common regulatory hubs for tolerance to acidic pH, Al and Pi-deficiency in *Arabidopsis* (Ojeda-Rivera et al. 2020). Finding orthologs for these regulatory genes in crops might prove a good starting point toward new targets for breeding and genetic engineering strategies.

### Major QTLs associated with low phosphate tolerance

Quantitative trait loci (QTLs) associated with enhanced Pi acquisition and utilization efficiencies have been described in several crop species including rice, maize, bean, rapeseed and soybean (Maharajan et al. 2018). The traits related to these QTLs include root length, lateral root number, root surface area, adventitious root number, lateral root diameter, and root and shoot ratio, among others (Maharajan et al. 2018). Only a few studies have been able to identify and validate the genetic features underlying QTLs related to root architectures that are better adapted to low-Pi soils. The QTL named *Phosphorus Uptake 1* (*Pup1*) confers Pi-starvation tolerance in rice variety Kasalath (Wissuwa et al. 2002). Further studies identified a protein kinase underlying *Pup1* QTL; this gene was named *OsPSTOL1* (*PHOSPHORUS STARVATION TOLERANCE* 1) (Gamuyao et al. 2012). The expression pattern of *OsPSTOL1* in low-Pi intolerant rice varieties indicates that it acts as an enhancer of early root growth by increasing the plant capacity to acquire more nutrients including Pi (Gamuyao et al. 2012). Interestingly, when introduced in Pi-starvation intolerant rice varieties, OsPSTOL1 significantly enhanced grain yield in Pi-deficient soils (Gamuyao et al. 2012). However, although the *PSTOL1* homolog in wheat contributes to agronomically important traits like flowering time and grain size, its manipulation did not show an increase in Pi use efficiency in this species (Milner et al. 2018). Moreover, *Pup1*-specific markers are not present in the rice landrace Wazuhophek, recently reported as low-Pi tolerant, indicating the potential use of this material as donor of novel loci for breeding programs (Swamy et al. 2019). Another approach for improvement of low Pi-tolerance in crops includes introgression. Wheat lines carrying wheat-rye chromosomal translocations showed a significant positive correlation between root biomass and Pi uptake (Ehdaie et al. 2010; Jung and Seo 2014; Moskal et al. 2021). Thus, suggesting this could be a promising tool for breeding low-Pi tolerance in other crops. We summarized major QTLs related to Pi-use efficiency and Pi-acquisition recently discovered in crops in Table 1 and encourage the reader to get further details for rice (Mahender et al. 2018), soybean (Kumawat et al. 2016; Zogli et al. 2017), maize (Wang et al. 2019a) and wheat (Colasuonno et al. 2021) studies.

**Table 1:** QTLs associated with phosphate-deficiency tolerance with promising potential for crop improvement

Plant species	Improved plant trait under low-P	QTL name	Reference
Rice (*Oriza sativa*)	Shoot weight, root weight, total weight	*qRST9.14*	(Mai et al. 2021)
	Plant height, shoot length, number of productive tillers, panicle length, dry shoot weight, root volume, yield, biomass, root/shoot ratio, seed P content	*qPH8, qSL8, qNPT8, qPL8, qDSW1, qDSW8, qRV7, qRV8, qSY8, qBiomass1, qBiomass8, qr1, qr8, qseedP1, qseedP7, qseedP8*	(Kale et al. 2021)
	Relative shoot dry weight, relative total dry weight, relative root length, relative root-to-shoot ratio	*qTPDE4*^*XB*^	(Zhang et al. 2018b)
	Dry shoot weight, dry root weight, total dry weight traits	*qRPUUE9.16*	(To et al. 2020)
Maize (*Zea mays*)	Root dried weight, P uptake efficiency, P usage efficiency, seminal root length, seminal root number, P usage efficiency, P uptake efficiency	*Cl-bin3.04a, Cl-bin3.04b*	(Gu et al. 2016a)
	Root length, root diameter, surface area of fine roots, root/shoot ratio	*qRL8.05, qRD1.03, qRD4.05, qRD7.02, qSA2_10.03, qRS1.07, qRS3.06*	(Azevedo et al. 2015)
	APase activity in root, APase activity in rhizosphere soil	*BBAPR2a, BBAPR3a, HCAPR2a, HCAPR3a, HCAPR4a, HCAPR9a, HCAPR2a, HCAPR5a, HCAPR7a, HCAPR8a,* *HCAPS1a-c, HCAPS3a, HCAPS5a, HCAPS9a-b, HCAPS10a*	(Qiu et al. 2014)
	H^+^ secretion	*nlg2228-bnlg100*	(Chen et al. 2011)
	Grain yield, leaf area, leaf length, leaf width, chlorophyll level, flowering time, anthesis silking interval	*qPGY4, qPGY5, qPGY7* *qPLA3, qPLA4, qPLA6, qPLA9, qPLA10, qPLL2-1, qPLL2-2, qPLL6, qPLW9, qPSPAD4, qPSPAD6, qPSPAD7, qPFT2, qPFT4, qPASI4*	(Cai et al. 2012a)
	Shoot dry weight, root dry weight, total root surface area, total root length, axial root length, axial root number	qSDW16-1, qSDW18-1, qSDW110-1, qSDW29-1, qSDW35-1, qRDW16-1, qRDW26-1, qRDW29-1, qRDW110-1, qRDW210-1*, qTRSA14-1, qTRSA16-1, qTRSA17-1, TRSA21-1, qTRSA110-1, qTRL11-1, qTRL14-1, qTRL16-1, qTRL17-1, qTRL19-1, qTRL21-1, qTRL110-1, qARL21-1, qARL22-1, qARL26-1, qARL28-1, qARL210-1, qARL35-1, qARL36-1, qARL310-1, qARN16-1, qARN110-1, qARN26-1, qARN210-1, qARN310-1	(Cai et al. 2012b)
Wheat (*Triticum aestivum*)	Plant height, spike length, spike number per plant, grain number per spike	*QPh-6D, QSl-7D.2, QSn-5A.1, QGn-7B*	(Yuan et al. 2017)
	Root dried weight, maximum root length	*qRDW.LP-4B, qMRL.LP-2B, qMRL.LP-5A, qMRL.LP-6B, qMRL.LP-7B*	(Ren et al. 2017)
	Rhizosheath size	*D_contig14507_369, Excalibur_c14216_692, Ex_c70232_336, GENE-2724_97, BobWhite_c8579_56*	(James et al. 2016)
	Rhizosheath size	*Tdurum_contig14482_423, wsnp_Ex_rep_c67296_65839761, IACX5879, BS00068710_51, IAAV7384, BobWhite_rep_c49790_351*	(Delhaize et al. 2015)
	Root length, root volume, root tip number, root surface area, shoot dry weight, root dry weight, total dry weight, ratio root to shoot dry weight	*QRL.caas-6BL, QRL.caas-7BL, QRL.caas-7AL, QRL.caas-7AS.2, QRV.caas-7AS, QRTN.caas-2BL, QRTN.caas-7BL, QRTN.caas-7AL, QRTN.caas-2DL, QRTN.caas-7AS, QRTN.caas-3DL, QROSA.caas-7AS, QSDW.caas-6BL, QSDW.caas-6DS, QSDW.caas-3AS, QSDW.caas-3DL, QRDW.caas-4BS, QRDW.caas-4DS, QRDW.caas-3AS, QRDW.caas-4BS, QTDW.caas-4BS, QRRS.caas-4BS.1, QRRS.caas-4DS, QRRS.caas-4BS.2, QRRS.caas-4DS*	(Yang et al. 2021)
	Biomass	*Wmc44, Barc045, Nw3071, Nw2245, Nw2703, Barc286, Wmc525*	(Ryan et al. 2015)
	Plant height	*QPh-2D, QPh-4B*	(Xu et al. 2014)
	Shoot length, thousand kernel weight, kernel weight per spike, total spikelet number per spike, sterile pikelet number per spike, harvest index, spikelet compactness, grain nitrogen concentration, straw nitrogen uptake, N utilization efficiency for grain yield, plant height, maximal root length, axial root number, stem and leaf dry weight, seedling dry weight, stem and leaf P utilization efficiency, root P utilization efficiency, shoot P utilization efficiency, stem and leave P content	*QSl-2D, QTkw-3B, QTkw-3B.1, QTkw-4D, QKws-6A, QTss-7A, QSss-2D, QHi-4B, QScn-2D, QGnc-6A, QSnup-5A.1, QNUtEGY-4D, QNUtEGY-6A,* *QPH-1D, QPH-3A, QPH-3B.2, QPH-5D, QPH-7B.1, QRL-1A, QRL-5B, QRL-7A, QRN-2B.1, QRN-2B.2, QSLDW-1D, QSLDW-4D, QSLDW-5D-1, QSDW-1D, QSDW-3B, QSDW-5D, QSLPU-2B, QSLPU-5A.1, QRPU-4B, QRPU-7B, QSPU-2B, QSLPC-2B, QSLPC-3A*	(Zhang and Wang 2014)
Soybean (*Glycine max*)	P use efficiency, root dry weight, total dry weight, plant height, P concentration	*q4-2*	(Zhang et al. 2016)
	Individual seed weight, intact pod weight, seed volume, seed protein	*SeedwtQTL4.1, SeedwtQTL4.3, SeedwtQTL6.1, PodwtQTL4.2, VolQTL6.2, ProtQTL20.1*	(Hacisalihoglu et al. 2018)
	100-seed weight	*qSW17-2,*	(Wu et al. 2020)
Barley (*Hordeum vulgare*)	Grain P concentration, straw P concentration, plant P concentration, grain P uptake, straw P uptake, plant P uptake, grain P utilization efficiency, straw P utilization efficiency, plant P utilization efficiency, grain yield, straw yield, dry matter	*Qgpc.sau-3H, Qspc.sau-3H, Qspc.sau-7H, Qpc.sau-3H, Qgpup.sau-1H, Qspup.sau-3H, Qpup.sau-1H, Qgpue.sau-3H, Qspue.sau-7H, Qpue.sau-3H, Qgy.sau-5H, 7H, Qsy.sau-3H, Qdm.sau-3H, Qdm.sau-5H, Qdm.sau-7H*	(Gao et al. 2020)
	Total root length, total root surface area, total root volume, adventitious root length, adventitious root surface area, adventitious root volume, lateral root length, lateral root surface area, lateral root volume, lateral root length	*Qtrl.sau-7H.01, Qtrl.sau-7H.02, Qtrl.sau-4H.01, Qtrsa.sau-4H.01, Qtrsa.sau-7H.01, Qtrsa.sau-4H.02, Qtrsa.sau-3H.01, Qtrv.sau-3H.01, Qarl.sau-4H.01, Qarl.sau-3H.01, Qarl.sau-3H.02, Qarsa.sau-4H.01, Qarsa.sau-3H.01, Qarv.sau-4H.01, Qlrl.sau-2H.01, Qlrsa.sau-4H.01, Qlrsa.sau-2H.01, Qlrv.sau-3H.01, Qlrl.sau-4H.01, Qlrl.sau-2H.01*	(Guo et al. 2018) (Gao et al. 2018)
Sorghum (*Sorghum bicolor*)	Grain yield, root diameter, surface area of fine roots between 1–2 mm in diameter	*Gy-1, Gy-3, Gy-4.1, Gy-4.2, Gy-6.1, Gy-6.2, Gy-6.3, Gy-9, SA2-3, SA2-8, RD-5, Gy/SA2-3, RD/SA2-2, Gy/RD-7*	(Bernardino et al. 2019)
Bean (*Phaseolus vulgaris*)	N fixation, yield components, phenological, photosynthetic	*%Ndfa7.1, TNdfa7.1, TNdfa8.1, TNdfs8.1, SdN2.1, SdN2.2, SdN7.1, SdN_ha 2.1, SdN_ha 7.1, SdCN2.1, SdCN7.1, PHI4.1, PHI5.1, PHI5.2, SBH2.1, SBH5.1, SBH11.2, SdC4.1, SdC6.1, Yd2.1, Yd7.1, Yd7.2, PNA2.1, PNA4.1, PNA4.2, PNA6.1, SdNA2.1, SdNA6.1, SdNA6.2, 100SdW2.1, 100SdW6.1, 100SdW6.2, 100SdW9.1, 100SdW9.2, DF4.1, DF5.1, DF5.2, DF5.3, DF5.4, DF11.1, DF11.2,, SCMR_f3.1, SCMR_f3.2, SCMR_f4.1, SCMR_f6.1, SCMR_f6.2, SCMR_f6.3, SCMR_m2.1, SCMR_m6.1, SCMR_m6.2, FVFM11.3*	(Diaz et al. 2017)

The studies described were limited to experiments carried out under greenhouse or field conditions

*P*: phosphorus, *N*: nitrogen

*These QTLs are changed according to developmental stage

## Strategies to produce low phosphate-tolerant plants

**Fig. 6:**
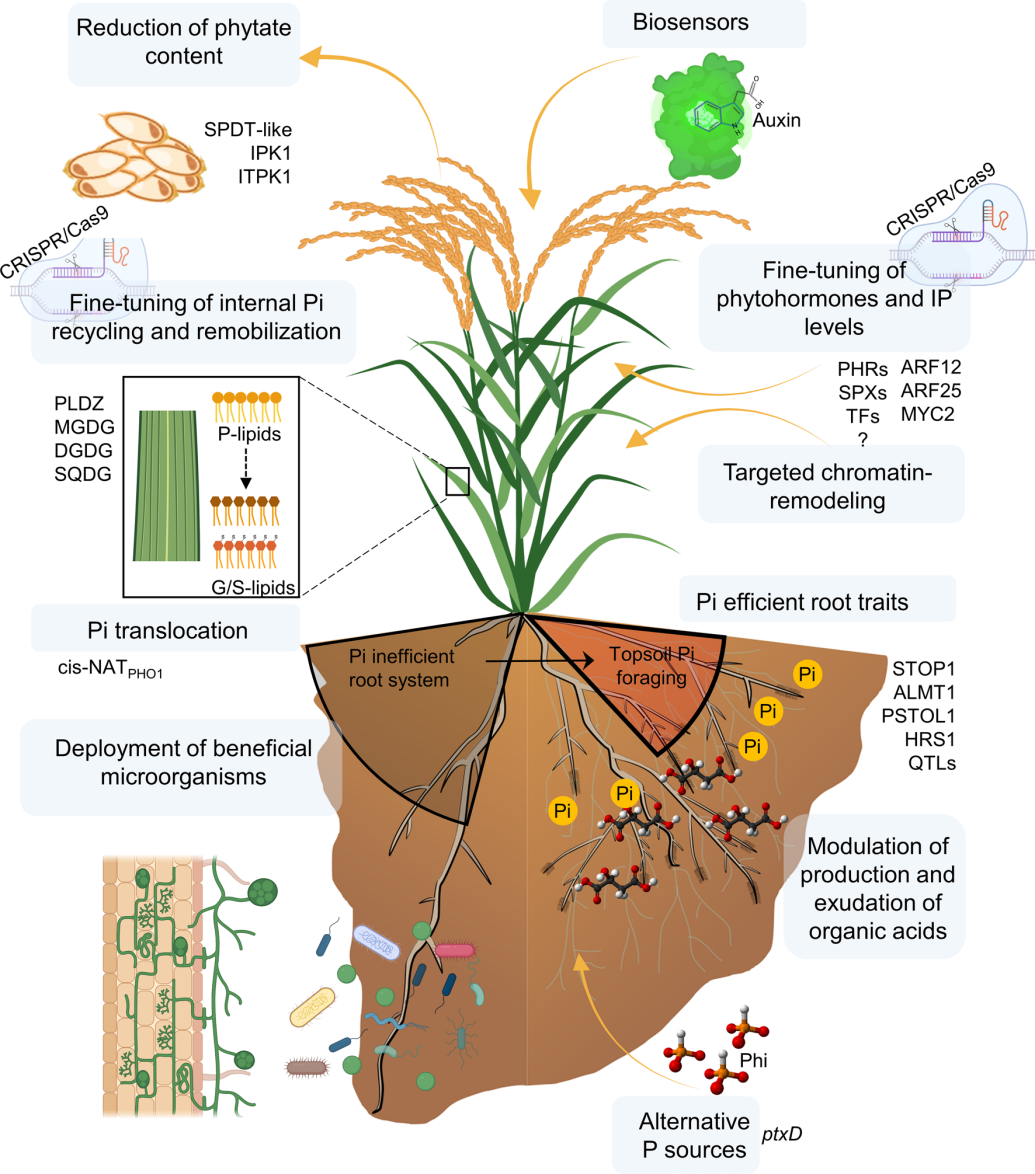
Overview of traits and strategies to develop low-phosphate (Pi) tolerance in crops. Several traits and strategies have potential to develop low Phosphorus (P) tolerant crops. These include strategies related to the deployment and engineering of plant associated microorganisms, the use of alternative P sources like phosphite (Phi), and the improvement of the plant genetics based on current knowledge of the molecular regulation of Pi starvation responses. This latter considers the fine-tuning of different traits such us Pi recycling and remobilization, phytohormones and inositol polyphosphate molecules (IP) levels and modulation of organic acids exudation. The use of the CRISPR/Cas9 technology together with more integrative synthetic biology approaches assisted by biosensor molecules could help speed up this process. Phospholipids (P-lipids), galactolipids (G-lipids), sulfolipid (S-lipids). Some elements in this figure were created with BioRender

Potential routes to accelerate the development of low-Pi tolerant crop varieties include favoring changes in root system architecture, improvement of Pi uptake and remobilization, remodeling of rhizosphere-microbial associations, and fine-tuning of organic acids secretion. In this section we discuss on the potential of some of these routes and propose novel strategies (Fig. 6).

### Genes for the modulation of phosphate metabolism

In the context of genetic engineering, the heterologous expression of bacterial genes to improve Pi-metabolism from alternative Pi sources seems very promising. Transgenic *Arabidopsis* plants expressing bacterial phytases (PaPhyC and 168phA) can use phytic acid as a Pi source without any negative physiological effects on plant development and germination (Valeeva et al. 2018). Furthermore, reduction of phytic acid content in grain has been also proposed as a solution that might contribute to reduce Pi-based fertilizer use by preventing excessive removal of Pi from the soil (Perera et al. 2018). This is the case of the *SULTR-like Pi DISTRIBUTION TRANSPORTER (SPDT)*, a sulfur transporter (*SULTR)-like,* that is expressed in the rice nodes and controls Pi allocation to the grain. Rice *spdt* knockout lines resulted in reduced phytate content in grain without compromising yield and seed germination (Yamaji et al. 2017). Likewise, knocking out *IPK1* (Inositol pentakisphosphate 2) and *ITPK1* (Inositol tetrakisphosphate 1) kinase-coding genes in crops such as maize, rice and wheat resulted in plants with reduced accumulation of phytic acid in the seed (Shi et al. 2003; Shukla et al. 2009; Ali et al. 2013; Aggarwal et al. 2018).

Engineered plants that express the *ptxD* gene from *Pseudomonas stutzeri,* which codes for an oxidoreductase that oxidizes phosphite into Pi, can use this compound as a P-source, requiring 30–50% less P input than when fertilized with the traditional P source under greenhouse (López-Arredondo and Herrera-Estrella 2012). This technology has been applied in rice, maize, and cotton plants that can be fertilized with phosphite as the sole source of P (Nahampun et al. 2016; Manna et al. 2016; Pandeya et al. 2018; Lopez-Arredondo et al. 2021). One of the main advantages of phosphite-fertilization technology is that it suppresses weed growth and enables transgenic crops to outcompete aggressive and glyphosate-resistant weeds while, at the same time, phosphite provides Pi-fertilization that can only be harnessed by *ptxD-*expressing plants (López-Arredondo and Herrera-Estrella 2012; Pandeya et al. 2018). Although these strategies seem feasible, applicable to several crops, and have proven effective under field conditions, as is the case of the *ptxD*/Phi technology, a long path for their optimization and implementation at large-scale still remains to be completed.

### Deployment of symbionts to enhance phosphate uptake

Plant roots host a diversity of bacterial communities which include Actinobacteria, Bacteroidetes, Firmicutes and Proteobacteria. These microorganisms provide protection from pathogen attack and enable nutrient foraging through solubilization (for review see Bulgarelli et al. 2013). Endophytic fungi are microorganisms that live within the plant and have been object of study for their possible role in the enhancement of plan nutrient acquisition (for review see García-Latorre et al. 2021). Root colonization by microbiota and endophytic fungi have proven beneficial roles in improving plant fitness under Pi-starvation in plants (Hiruma et al. 2016; Castrillo et al. 2017). In *Arabidopsis*, this process is mediated by AtPHR1 (Hiruma et al. 2016; Castrillo et al. 2017). Thus, fine-tuning of PHR1-mediated regulation in crops also emerges as a tool to enhance root colonization by beneficial microbial consortiums. Inhibition of suberin deposition by microbiota facilitates plant nutrient uptake (Salas-González et al. 2021). This process occurs via the inhibition of abscisic acid signaling (Salas-González et al. 2021), thus suggesting the downregulation of synthesis-related genes in crops to prevent suberization and promote association with beneficial consortiums of rhizosphere microbiota. Inoculation of root crops with Pi-solubilizing microorganisms is also an interesting solution to enhance Pi availability from organic matter in soil (Alori et al. 2017).

Root symbiosis with AMF is another type of beneficial symbiotic association. It may benefit Pi uptake for the plant in exchange for carbohydrates for the fungi and contribute to improve plant Pi nutrition (Willmann et al. 2013; Liu et al. 2018a; Chen et al. 2018). AMF are effective in foraging Pi for the plant as these symbionts can spread beyond the rhizosphere Pi-depleted zones and provide Pi that otherwise would be inaccessible for the root system (Chen and Liao 2017). The majority of plant species (~ 70%) form symbiotic relationships with AMF including crops of major economic importance like maize and rice. Nonetheless, there are some exceptions which include the Chenopodiaceae, Carophyllaceae, and Brassicaceae plant families (Cosme et al. 2018). The latter includes some crops of agricultural relevance like cabbage and broccoli and the model plant *Arabidopsis*. In the case of maize, for instance, AMF-symbiotic Pi uptake accounts for up to one third of grain yield in rain-fed sub-tropical fields (Ramírez-Flores et al. 2020). Up to 70% of Pi-uptake in rice is symbiotically acquired, corroborating the relevance of AMF in crops (Yang et al. 2012). Pi-transporters induced by AMF in rice (OsPT11/13) and soybean (GmPT7/10/11) are evidence of plant Pi-uptake at the symbiotic interface (Chen and Liao 2017). Interestingly, high Pi fertilizer application can reduce the percentage of root colonization and decrease AMF biomass (Smith et al. 2011; López-Arredondo et al. 2014) which indicates that AMF might only play a Pi-uptake enhancing role when Pi conditions are limiting. Further investigation on the role of AMF in “high Pi” soils is required to sort out this controversy. Moreover, knowing the genetic elements that regulate the activation/deactivation of the symbiotic Pi-uptake pathways could lead to the development of plant varieties that could maintain AMF symbiosis independently of the Pi status in the plant. Undoubtedly, because limited Pi-availability is a major constrain in most croplands, deployment of AMF remains a valuable source to enhance crop Pi uptake.

### The potential of genome editing-approaches

CRISPR/Cas (Clustered Regularly Interspaced Short Palindromic Repeats/CRISPR-associated protein) has emerged as one of the most promising systems to facilitate crop improvement by altering in a precise way the genome of any organism (Khatodia et al. 2016; Wang et al. 2019d; Zhao et al. 2019; Jiang and Doudna 2017). It offers a series of advantages over traditional approaches; for example, in contrast to the transgenic approach in which DNA expression cassettes are randomly inserted in the genome and may cause pleiotropic phenotypes, genome editing approaches allow a precise *design* of the desired trait. Moreover, since edited varieties may be generated via selectable marker-independent methods, they might be subject to less regulatory issues compared to transgenics. Thus, the resulting events may be directly incorporated into breeding programs and commercialized as feed and food.

The CRISPR/Cas system has been adapted to allow simultaneous editing of multiple sites of both coding and regulatory sequences. Furthermore, the availability of multiple Cas proteins (e.g., Cas9, Cas12, Cpf1, CasΦ) and Cas9 variants [nickase Cas9 (nCas9), deactivated Cas9 (dCas9)] expands the application of the CRISPR/Cas system beyond editing (Jaganathan et al. 2018). Besides the “off-targets” mutations that it may induce and some technical hurdles for its successful implementation (see Montecillo et al. 2020), the CRISPR/Cas system has boosted the study of plant responses to diverse abiotic stresses including drought, and K and N deficiencies and to a lesser extent P starvation. Most of the studies have been performed specially on rice, maize, and tomato and are limited to the functional validation of already reported genes in which knockouts are described, thus providing the proof-of-concept for future crop improvement (Jaganathan et al. 2018). Using CRISPR/Cas, deletion mutants of the AtPHO1 homolog in tomato, *Sl*PHO1;1 were generated, which displayed typical characteristics of Pi-starvation, shorter and redder leaves than the wild-type control as previously reported for *Arabidopsis* (Zhao et al. 2019). Allelic variants of HvITPK1 in barley were also generated with the purpose of lowering phytic acid levels (Vlčko and Ohnoutková 2020). Interestingly, barley ITPK1 allelic variants showed salinity stress tolerance, thus posing new questions on the role of these enzymes on general abiotic stress signaling. A similar approach was implemented using TALEN and CRISPR/Cas9 to lower phytic acid synthesis by impairing *ZmIPK1* in maize (Shukla et al. 2009; Liang et al. 2014). In a recent report, the role of OsACS1 (1-aminocyclopropane-1-carboxylic acid synthase) and OsACS2, involved in ethylene biosynthesis, was investigated in rice by taking advantage of CRISPR/Cas9 (Lee et al. 2019). Both enzymes were found involved in remodeling *Arabidopsis* root system architecture, transcriptional regulation of Pi-starvation responsive genes, and Pi homeostasis.

Evidence  on N metabolism provide clear examples of the utility and effectiveness of this technology, thus suggesting it is a plausible strategy to speed up targeted crop breeding for low-Pi tolerance. An interesting case study is the NRT1.1 nitrate transporter in rice. Previously, a single nucleotide polymorphism consisting of C/T (Thr327Met) change was associated with N-use efficiency improvement (Hu et al. 2015). Therefore, by using and optimizing the CRISPR/Cas9 system, the precise base-editing to generate C/T replacement at this location (Thr327Met) in NRT1.1B was successfully done (Lu and Zhu 2017). Furthermore, Li and co-workers (2018), using CRISPR/Cas9 replaced the complete japonica NRT1.1B allele by the high N-use efficiency indica allele in only one generation (Li et al. 2018), which by traditional introgression would take between 4 and 6 generations. These examples prove the versatility and power of this technology to improve agricultural traits.

### Transcriptional engineering

Transcription factors represent promising candidates for improving resistance to multiple environmental stresses. Through controlling growth and development, transcription factors orchestrate plant responses to various abiotic stresses and present overlapping functions (Patikoglou and Burley 1997; Lindemose et al. 2013). Several transcription factors belonging to different families have been described to play crucial roles in modulating low-Pi starvation responses and crosstalk with hormones in crops including MYB62 (regulating the crosstalk between Pi and gibberellic acid) (Devaiah et al. 2009), ETC1 (important in favoring higher root hair density under low-P conditions) (Savage et al. 2013), and MYB2 (involved in mediating Pi-starvation responses through miR399f) (Baek et al. 2013; Jyoti et al. 2019). Thus, opening new avenues to apply transcriptional engineering approaches by means of engineering multiple transcription factors simultaneously.

In recent years, regulatory networks composed of key transcription factors controlling plant responses to Pi-starvation have been identified in rice which include a network of 266 transcription factors underlying low-Pi responses in the context of AMF symbiosis (Shi et al. 2021). The network is centered in PHR transcription factors, widely known to control Pi-starvation adaptive responses (Fig. 4a), and is enriched in Pi-starvation, N-metabolism, and chromatin remodeling related transcription factors. Moreover, it captured transcription factors controlling mycorrhizal symbiosis and hormone signaling pathways (i.e., ethylene, jasmonic acid, auxins) reported previously such as ARF12, ARF25, MYC2, among others (Shi et al. 2021). These findings represent emerging opportunities to modulate and remodel in a precise way the activity of specific nodes of regulation orchestrating low-Pi responses at specific organs or cell types, and at specific developmental stages when the plant requires Pi the most. Such level of regulation involving a crosstalk between Pi metabolism, mycorrhizal symbiosis, and hormone signaling, suggests that highly complex and intricate regulatory mechanisms underlie Pi-nutrition in the context of biotic interactions. This complexity can be studied by applying CRISPR/Cas multiplexed approaches. These studies should allow genome-wide transcriptional reprogramming in crop plants for breeding purposes. Gene regulons composed of key transcription factors, protein regulators, and microRNAs controlling Pi homeostasis in plants are presented in Fig. 3, which can serve as the basis to remodel Pi-starvation responses in crops.

An interesting candidate is the transcription factor PHR1. Three residues (K325, H328, R335) in the coiled-coil (CC) domain of AtPHR1 are essential for binding AtSPX1 (Ried et al. 2021). Interestingly, mutation of these residues disrupts AtSPX-binding without affecting AtPHR1’s ability to bind its target sequence which results in constitutive activation of Pi-starvation responses (Ried et al. 2021). Now, with the possibility of doing specific nucleotide base replacements with CRISPR/Cas9, strategies to study these three PHR1^KHR^ residues can be implemented. Although this task might be technically challenging, it could be interesting to assess how the replacement of these residues impacts the low-Pi stress responses in crops. Likewise, IP molecules are essential to stabilize the structure of SPX proteins, enabling them to interact with regulators of Pi homeostasis in eukaryotes (Wild et al. 2016). The possibility of fine-tuning IP-molecules biosynthesis, IP_6_, IP_7_ and IP_8_ for instance, remains an interesting perspective for future breeding. Given the essential role of IP molecules as cofactors of auxin and jasmonic acid sensing complexes and the crosstalk with plant Pi status, a possibility remains that by modifying the balance of IP-signaling molecules such as IP_8_, IP_5_, and IP_6_, stress responses can be finely modulated at a system level as well.

### Fine-tuning of plant hormones and organic acids

Due to the crosstalk between hormone signaling and the regulation of Pi-starvation responses, a tight modulation of these responses can be achieved by fine-tuning both internal and secreted hormone levels through CRISPR/Cas9. Strigolactones are hormones involved in regulating the growth of primary and lateral roots; upregulation of strigolactone synthesis and root-exudation under Pi-limiting conditions have been shown to favor AMF symbiosis (Mayzlish-Gati et al. 2012; Sun et al. 2014; Santoro et al. 2020). However, high levels of strigolactones can also promote the germination of weeds, for example *Striga*, which threatens crop productivity (Khosla and Nelson 2016; Yacoubou et al. 2021). Therefore, by fine-tuning strigolactone levels, through generating CRISPR/Cas9 allelic variants, one would be able to improve crop fitness by modulating root traits and AMF symbiosis in benefit of low-Pi tolerance while avoiding weeds germination. CRISPR/Cas9 edited rice lines to disrupt CCD7 (*CAROTENOID CLEAVAGE DIOXYGENASE 7*), an enzyme involved in a limiting step in strigolactone biosynthesis, have already been reported (Butt et al. 2018). Interestingly, CCD7-edited lines showed reduced height, increased tiller production, and reduced exudation of strigolactones compared to the wild-type. However, total yield, plant Pi-starvation responses, and association with AMF remain to be studied in these plants. Further studies are required to test the effect of strigolactone downregulation in AMF formation and Pi nutrition in soils with Pi limitation.

The transcription factor STOP1 controls an interesting network that modulates root adaptation to acidic soils, which present low pH, Al toxicity and low-Pi availability (Sawaki et al. 2009; Ojeda-Rivera et al. 2020). Some of the STOP1-targets include organic acid transporters that mediate Pi solubilization and, in the particular case of *ALMT1*, mediate root growth in response to low-Pi availability (Balzergue et al. 2017; Mora-Macías et al. 2017). Because the STOP1-binding site has been identified and the activation of STOP1-targets correlates with its nuclear accumulation (Ojeda-Rivera et al. 2020), the CRISPR/Cas9 system could be used to edit the promoter sequence of STOP1-target genes to modulate and re-wire root organic-acid synthesis and exudation. This strategy might contribute to designing crops that are able to thrive and have a root system architecture better adapted to acidic soils with low-Pi availability. In this context, another interesting candidate gene is the root specific HRS1 (hypersensitivity to low Pi-elicited primary root shortening 1) transcription factor, involved in modulating primary root growth in response to Pi-starvation only in presence of nitrate (Liu et al. 2009; Medici et al. 2015). Therefore, as this transcription factor helps integrate signaling responses to both nitrate and Pi signals in the root tip (Medici et al. 2015), it provides an interesting perspective to achieve the coordinated modulation of plant responses to both stresses.

Fine-tuning of Pi recycling and remobilization has been proposed as a key breeding target for producing Pi-use efficient genotypes because it could enable plants to release and mobilize Pi from phospholipids without compromising photosynthesis (Heuer et al. 2017). Genomic and transcriptomic characterization of gene-regulation networks in the Proteaceae family, which adapted to environments with extreme Pi-limitation, should provide insights into the re-wiring of phospholipid metabolism (Hayes et al. 2018). Editing of promoter sequences of genes related to phospholipid substitution without compromising photosynthesis in crops, seems an interesting strategy.

## Perspectives

Current agricultural systems are built on improved crop varieties and the extensive use of agrochemicals. For years, breeders have taken advantage of natural or induced genetic diversity in order to generate improved varieties. Clear examples of these efforts can be seen in improved rice varieties that have undergone drastic changes in plant architecture and grain yield and, thus, have sustained the Green Revolution. However, this process is slow and its application on crop low-Pi tolerance has not yet produced the varieties needed to significantly reduce the application of P-fertilizers. By exploiting the promises of novel approaches such as CRISPR/Cas9 and synthetic biology it would be possible to speed up the creation of the genetic diversity that is required for breeding purposes, and even to enrich this diversity beyond what we naturally though possible.

Now that complex networks of regulation controlling Pi-starvation responses are being elucidated and versatile CRISPR/Cas-based gene editing strategies can be implemented with the help of the latest and more sophisticated on-development computational resources, we might think on crop improvement based on more rational designs that can be systematically generated and evaluated (Liu and Stewart 2015). In this context, synthetic biology approaches that integrate and take advantage of multiple disciplines for the redesign and rewiring of genetic connections might be a useful asset (Kassaw et al. 2018; Pouvreau et al. 2018). This will help removing inefficiencies naturally associated with these processes while optimizing the essential components toward the desired trait. As stated by Medford and Prasad (2014), this type of approaches will help “not only uncover the natural genetic circuits behind complex gene regulations in plants, but we could also design traits that are new to evolution and beneficial to humanity” (Medford and Prasad 2014).

The design of biosensor molecules might also contribute to achieve these types of strategies by adding an external predictable and controllable switch to the system. The infiltration into plant cells of nanosensors wrapped into single-walled carbon nanotubes has already been used for different purposes including detection of external nitroaromatic compounds (Wong et al. 2017), arsenic (Lew et al. 2021), auxins (Ang et al. 2021), and H_2_O_2_ in stressed plants (Lew et al. 2020). Furthermore, a genetically encoded auxin biosensor was recently reported that allows the direct, rapid and real-time monitoring of auxin levels in individual cells and within cell compartments during the plant´s life cycle (Herud-Sikimic et al. 2021). This sensor is based on the rational engineering of a tryptophan repressor from *E. coli* and although originally designed for *in planta* visualization of auxin concentration, it can be easily incorporated for the design of biotechnological applications.

The previously mentioned perspectives can be accomplished by thoroughly harnessing all the power and potential from emerging technologies. Short- and long-read sequencing technologies should coordinately aid in deciphering the genomes and transcriptomes of species adapted to extreme Pi-scarcity and provide new gene pools and gene regulatory networks for low-Pi tolerance. Single-cell technologies will be crucial to decipher the hierarchical role of molecular regulators in such gene networks. Developments in proteomics, like the recent application of nanopores to determine protein fingerprinting (Lucas et al. 2021), together with high-throughput metabolomic characterization should aid defining the phenotypic profile of the Pi-efficient crop varieties. The development of efficient and standardized tissue-culture-independent gene editing systems will be key to speed up gene replacement and genome editing of crop plants which will ultimately help plant scientists and breeders to fine-tune plant Pi metabolism and root systems to boost Pi-foraging and Pi-use efficiency. Artificial intelligence approaches, like machine learning (Esposito et al. 2020), could be applied to detect and predict low-Pi tolerance-related genes and traits. Close collaboration of the scientific community and plant breeders with industry, policy and decision makers and government agencies must be promoted to guarantee technology transfer, a favorable public perception and fair distribution of crop materials to ultimately help us achieve sustainable crop Pi-nutrition.
